# Eye movement patterns drive stress reduction during Japanese garden viewing

**DOI:** 10.3389/fnins.2025.1581080

**Published:** 2025-05-15

**Authors:** Seiko Goto, Hiroki Takase, Keita Yamaguchi, Tomoki Kato, Minkai Sun, Aoi Koga, Tiankai Liang, Isamu A. Poy, Karl Herrup

**Affiliations:** ^1^Faculty of Environmental Science, Nagasaki University, Nagasaki, Japan; ^2^Division of Psychology, Faculty of Arts, Shinshu University, Matsumoto, Japan; ^3^Graduate School of Global Environmental Studies, Kyoto University, Kyoto, Japan; ^4^Graduate School of Art Studies, Kyoto University of the Arts, Kyoto, Japan; ^5^School of Architecture and Urban Planning, Suzhou University of Science and Technology, Suzhou, China; ^6^Center for Information and Communication Technology, Nagasaki University, Nagasaki, Japan; ^7^JACOBS School of Engineering, University of California, San Diego, La Jolla, CA, United States; ^8^Department of Neurobiology, University of Pittsburgh School of Medicine, Pittsburgh, PA, United States

**Keywords:** Murin-an, pulse rate, EMDR, eye tracking, visual stimuli

## Abstract

**Aim:**

The aim of this study is to clarify the role of eye movements in the reduction of physiological and psychological metrics of stress during Japanese garden viewing.

**Methods:**

We chose the well-structured Murin-an garden as a test site and a garden with similar visual elements but less well-maintained as a control site. We measured pulse rates and eye movements to monitor physiological responses. Psychological responses were tracked with the POMS2 Brief form and a short questionnaire.

**Results:**

We found that the Murin-an garden was more effective in decreasing pulse rate and improving mood. Also, in the Murin-an garden the participants’ gaze ranged more broadly across the visual field and moved more rapidly. Contrary to our expectations, in neither garden did pulse rate rise or fall based on the particular object a participant was viewing.

**Conclusion:**

Visual stimuli of a well-designed garden can elicit significant stress reduction. Our data suggest that the composition of the elements and the attention to maintenance of a garden result in viewers shifting their gaze more frequently and more quickly. These appear to be the key drivers behind the stress reduction effect. Although we had hypothesized that specific visual elements in the garden would be responsible for reducing measures of stress, our data instead suggest that it is the overall pattern of rapid horizontal eye movements, induced by the garden design, that drives the observed stress reduction. We draw an analogy between our results and the technique known as EMDR (eye movement desensitization and reprocessing) whose practitioners use rapid gaze shifts to elicit stress reduction.

## 1 Introduction

One traditional Japanese garden style is known as an “observation garden.” Unlike most gardens where a visitor is expected to move through its space and appreciate its elements from many different visual perspectives, an observation garden is designed to be viewed while seated at a single vantage point. Indeed, the location of this vantage point is a critical design feature that is carefully chosen to maximize the impact of the visual scene. One reason for imposing this level of control on the viewer is that observation gardens were meant to be more than merely esthetically pleasing. For example, they were favored within Zen temples as aids for meditation and thus performed an important function for the temple residents. Today, in addition to their original functions, many observation gardens have become tourist attractions. While their esthetic beauty is part of their appeal, it is likely that another important draw is their documented history of providing a calming effect on the viewer. The centuries of garden designers who perfected the design of the observation garden no doubt honed their skills by trial and error. Yet from a modern perspective the consistent impact of their creations on a visitor raises questions as to what might be the underlying physiological and neurobiological mechanisms that these architects tapped into.

The effect of a natural environment on human physiology has been noted both anecdotally and in formal research studies. Ulrich was one of the first modern practitioners of the latter approach (Ulrich, 1984). His idea was that “most natural views apparently elicit positive feelings, reduce fear in stressed participants, hold interest, and may block or reduce stressful thoughts; they might also foster restoration from anxiety or stress.” Soon after this work was published, Kaplan & Kaplan in Attention Restoration Theory (ART), proposed that unconscious mild esthetic experiences in nature can have a psychological healing effect (Kaplan, 1994; Kaplan and Kaplan, 1989). Other sensory stimulations have also been found to have positive health effects (Abraha et al., 2017; Blackburn and Bradshaw, 2014; Goris et al., 2016; Matthews, 2015; Moyle et al., 2014; Ohman et al., 2014; Vink et al., 2013). Works such as these open a critical window into the impact of the sensory environment on a person’s physiological state, yet most lack a strong neurobiological explanation. As the field progressed, and imaging technology became more widely used, we now appreciate that receiving stimulation from a visual field such as a garden is routinely associated with activation of specific regions of the brain.

Though the data is less extensive, there are increasing numbers of studies reporting on eye-movement activity during a visual stimulation involving a natural scene such as a garden (Qiu et al., 2023; Xu et al., 2024; Mohamed et al., 2019). Our own work has focused on studies such as these. What we have repeatedly observed is that when an individual views a structured natural space, created using the design principles of a Japanese garden, they tend to take in the scene presented with more visual fixation points and longer fixation times, than when viewing other types of green spaces (Sun and Fujii, 2018). As one example, the average duration of a fixation was 50% greater when viewing a Japanese style garden than when viewing a similar green space (Goto et al., 2018). The parasympathetic nervous system, which is associated with relaxation, was also found to become dominant when the participants’ eyes move back and forth across a Japanese garden (Liu et al., 2020).

**Figure 1:**
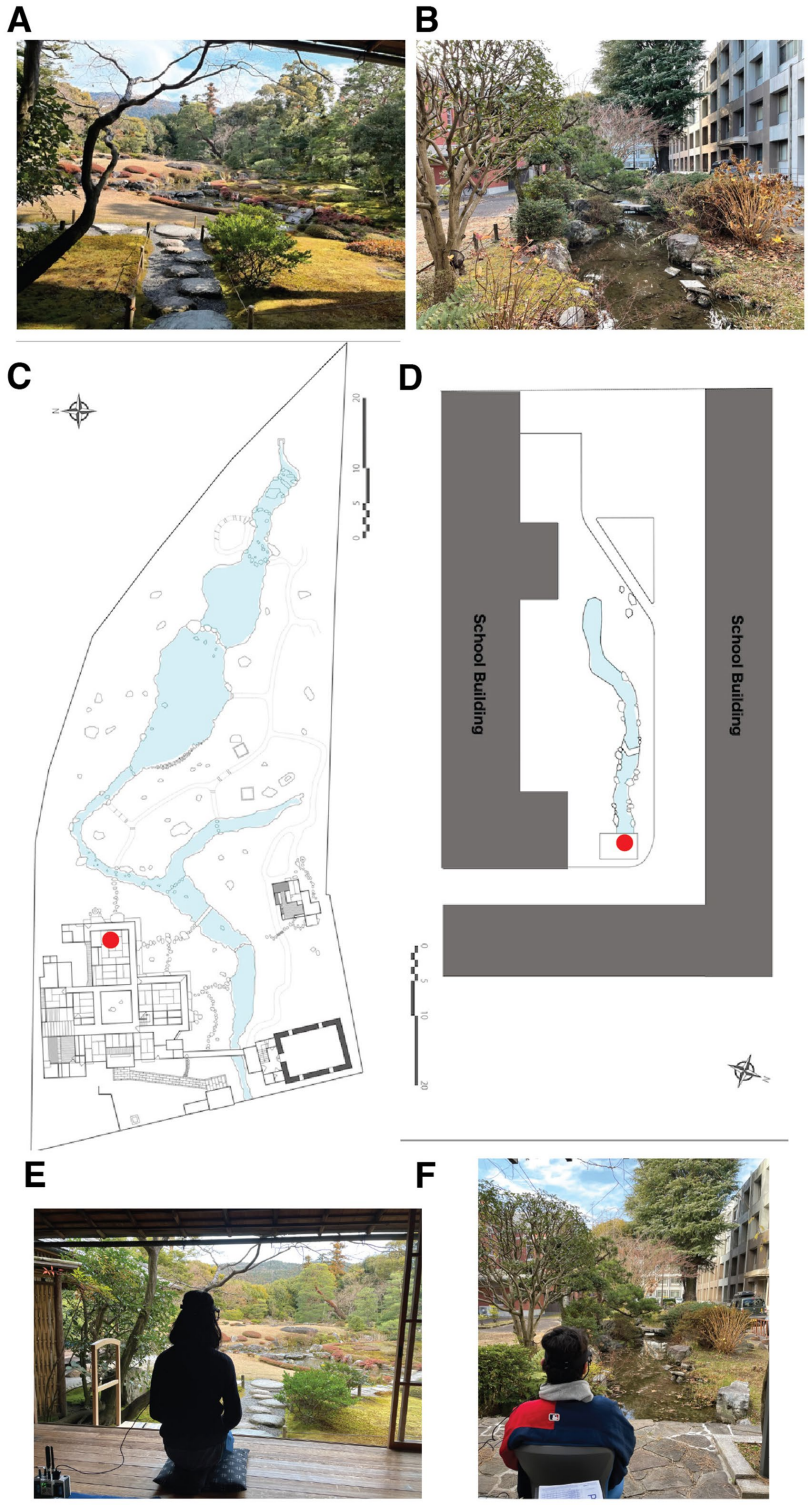
The two gardens used in the study. **(A)** Photograph of the Murin-an garden on the day of the experiment. **(B)** Photograph of the Kyoto University Garden (KUG) on the first day of the experiment. **(C)** The site plan of the Murin-an garden as provided by Tomoki Kato. **(D)** The site plan of the Kyoto University garden (provided by Tiankai Liang). **(E)** A photograph of a subject’s viewpoint of the Murin-an garden. **(F)** A photograph illustrating a subject’s viewpoint of the Kyoto University Garden.

Based on these results, we initiated the current study to test the concept that while the quality of the garden does indeed help shape its effectiveness in reducing stress in the viewer, it is the eye movements during a viewing period—the number of gaze points, and the speed with which the viewer shifts from point to point—that are important influences governing the magnitude of the response. We thus chose to assess the impact of a world-class Japanese garden, Murin-an, designed by one of the leading landscape architects of his time on a viewer’s level of stress. The Murin-an garden (Figure 1A) is located in the heart of Kyoto, Japan and has been designated as a historic site and place of scenic beauty by the Japanese government. It was originally built in 1894 as a villa for Yamagata Aritomo, the former prime minister of Japan. It was designed as an observation garden to be viewed from the center of the main room of the villa. The view takes advantage of a technique known as “borrowing scenery” by incorporating the nearby Mt. Higashi into the design (Supplementary Figure 1). The garden is meant to represent a river flowing from a distant mountain, with a shallow pond and zig-zag stream surrounded by lush vegetation. This peaceful atmosphere has earned the garden a reputation as being one of the most relaxing in the region. It has maintained this status due in large part to the fact that its maintenance is impeccable. This applies not just to the cleanliness of the various features but also to the rigorous attention to maintaining the shapes and spatial balance among the various trees and other plantings. We wished to ask how a viewer’s response to a world-class observation garden such as this would compare with their response to a Japanese style garden that was less rigorously maintained.

For this second type of garden, we chose one on the main campus of Kyoto University (Figure 1B). The garden is in the courtyard behind the civil engineering department building. The precise year of the garden’s construction and its designers are unknown. The garden features a stream at its center, with pine trees, shrubs, rocks, and a bridge carefully arranged around it. The garden also has a Himalayan cedar tree located at the edge of the stream, which was planted in 1912 by Sakuro Tanabe, a former dean of the school to commemorate the visit of Albert Einstein. At the end of the stream, a gazebo with a bench offers visitors a place to observe the garden. The most significant difference between the two gardens is that the space in Murin-an is enclosed by trees, whereas the garden at Kyoto University is enclosed by the university buildings. Our analysis of the response of subjects to these two gardens reveals that rather than one visual element driving the relaxation effect, it is the overall design features that work together to encourage a more holistic viewing strategy that is the key. We discuss the implications of this conclusion on the therapeutic value of other types of sensory stimuli.

## 2 Methods

Our experiments took advantage of a rare opportunity to have subjects view the two gardens with no distraction caused by the presence of other people during the observation period. We were particularly fortunate because the Murin-an garden is designated as a cultural property and had never been used for research purposes. It is a very popular tourist garden; between 2016 and 2018 there were more than 3,000 visitors per month. The garden is open to the public year-round, except for administrative and weather-related closures. This presented a challenge to us as it was almost impossible to schedule a large enough block of time to complete our experiments without the potential distractions of the normal crowd of tourists. We were fortunate to be able to negotiate with the Kyoto city government to find a mutually acceptable time. We then coordinated with the garden’s caretakers and staff to identify a single day in January when the garden was closed for its annual maintenance when we could perform our observations. While we had unfettered access to the facility, the lack of heat in the viewing room was an environmental factor over which we had no control. The second garden was located on the campus of Kyoto University. Here too we were fortunate in our experimental design in that during the same week that Murin-an was closed for maintenance, it was exam week at the University, which meant that the area surrounding the Japanese garden was virtually empty, with little or no pedestrian traffic to distract our subjects.

From a visual point of view, the most significant difference between the two gardens is that whereas the plants in Murin-an are carefully adjusted in size to maintain the balance of the overall landscape, in the garden at Kyoto University, the Himalayan cedar has been allowed to grow naturally without pruning. In Murin-an, the garden is meticulously cleaned, and the size of the plants are carefully adjusted to maintain the one point perspective-like view. The branches of the foreground trees are thinned out, allowing viewers to see the middle and distant scenery through the branches. The central part of the garden is open, and the stream of water runs diagonally. In contrast, the maintenance of the garden at Kyoto University has been lackluster. The overall landscape lacks balance, and the view behind the cedar is entirely blocked by its dense foliage. There are visible weeds and scattered litter throughout the space. The central part of the garden is filled with shrubs, and the stream of water runs straight from the viewer’s perspective in the direction of the cedar tree.

The observations were conducted over a three-day period in January during which time all 16 participants viewed both the Murin-an and Kyoto University gardens from the same location in either the Murin-an viewing room (red dot, Figure 1C) or from a comparable position in front of the Kyoto University garden (red dot, Figure 1D). A typical view from behind the subject during a viewing session is shown for Murin-an in Figure 1E and for the Kyoto University Garden in Figure 1F. The two gardens are both Japanese-style gardens centered around a stream, with spaces composed of the same visual elements: plants, rocks, lanterns, and steppingstones. The primary plantings in both gardens are pine, maple, and azalea, and the plants in both are shaped using traditional Japanese pruning methods.

### 2.1 Participants

As traveling to the two sites would have been more challenging for older, less mobile individuals, we recruited young, healthy college students for our experiments. Previous work has shown that all ages are impacted (Liu et al., 2020; Goto et al., 2020; Goto and Fritsch, 2011; Goto et al., 2014), lending confidence in the wider applicability of our findings. The total number of participants was 16, which was the maximum number we could use given the need to conduct our experiments in a single day at Murin-an. Among the 16 participants who took part in the study, 7 were undergraduate students from Kyoto University Department of Civil Engineering—5 males and 2 females—and 9 were undergraduate students from Kyoto Art University Department of Environmental Design—1 male and 8 females. The study participants were all between the ages of 21 and 24 years. All Kyoto University students were majoring in city planning and all the art students were majoring in Japanese garden design. All participants were physically healthy with good eyesight (uncorrected vision or soft contact lens correcting no more than 0.7 in the decimal system, approximately 20/32). We did not collect any other health or psychological data from the students.

All study methods were reviewed and approved by the Nagasaki University Research Ethics Committee, and we performed the experiment in accordance with our approved plan. The Committee is tasked by the University with assuring that all clinical research is conducted in accordance with the principles of the Helsinki Declaration (including the latest amendments) and other guidelines related to ethics in the field of research. Subjects were recruited between December 1, 2022, and January 16, 2023. Written informed consent was obtained from each subject prior to the initiation of the experimental protocol.

The subjects had two different educational interests. The nine art students from the Kyoto Art University studied Japanese gardens as part of their curriculum. The seven Kyoto University students were engaged primarily in policy and city planning courses and had limited detailed knowledge of Japanese gardens. Because it was on their campus, all policy/city planning students were familiar with Kyoto University Garden (KUG) while the art students had mostly never seen it before. All policy/planning students visited Murin-an first and all art students visited KUG first. Thus, as part of the study design, all participants first visited a garden that they had never seen before.

### 2.2 Materials

#### 2.2.1 Spatial measurements

The dimensions of the two gardens were similar. The Murin-an garden (Figure 1A) measured approximately 85.5 m in depth and 36.6 m in the perpendicular direction (Figure 1C) while the Kyoto University courtyard where the garden was located (Figure 1B) measured approximately 84.5 m in depth and 32.0 m in the perpendicular direction (Figure 1D). The location of the subject during the garden viewing is indicated by a red dot in Figures 1B,D. From this location, the elevation angles were similar: Murin-an 9.9° and KUG 8.2°. Because the viewing point of the Murin-an garden was bounded by the structural features of the room, the horizontal angle of Murin-an was confined to 23.68°, while the KUG horizontal angle was 45.29°.

#### 2.2.2 Spatial assessment

The space of the two gardens was surveyed using a VZ-400 3D laser scanner produced by RIEGL, plus a Canon E0S 600D camera with a Sigma 4.5 mm F2.8 EX DC Circular Fisheye lens. The VZ-400 uses a pulsed laser beam to scan the environment and generate a detailed 3D model. To ensure accuracy, the laser beam was positioned at the observer’s eye level (angle pitch 0.1°), and a 3D point cloud data was obtained with 5 mm precision.

#### 2.2.3 Eye-movement

To record eye tracking and fixation, we utilized the EMR-10 eye-mark recorder (NAC Image Technology Co., Ltd. Japan), a device that captures a high-speed video of eye movements using infrared light. It is non-invasive and uses a baseball cap with a small camera attached to the participant’s head via a headband. The camera records the eye positions and pupil movements. As the EMR-10 is designed to allow for head movement, participants can look around the garden with no restriction. To assure reproducibility, we recalibrated the instrument before each recording session. To track the eye positions and pupil movements over time, the recorded data were analyzed using the company’s software, which aggregates gaze images within a specified time, converts them into a heat map, and automatically produces Excel files containing fixation information. The EMR-10 considers periods with small variations in gaze (default 50 ms) to be a gaze point and assigns absolute coordinates. By default, the measurement data outputs the gaze point coordinates (X, Y, Z) of both eyes, the pupil diameter (mm) of both eyes, and the relative value of the pupils of both eyes to a CSV file every 17 msec. Additionally, the EMR-10 creates a heat map by performing automatic coordinate matching on the selected visual field image with image data within the specified time period and superimposes the data on the visual field image. In this study, the heat map of the cumulative data of the gaze points was constructed with the range set to 50 pixels, the dot strength set to 50, and the afterimage set to 10 s.

To quantify how long the viewer needed to assess the object during any one glance, we calculated the value known as the density of visual information (DVI), using following formula:

$\frac{\mathrm{fixation duration in}\;AOI}{\mathrm{total fixation duration}}$ $÷\frac{\mathrm{pixels in}\;AOI}{\mathrm{total pixels}}$

(where AOI = area of interest)

To measure how often the viewer focused on the same object, consciously or unconsciously, during the viewing session, we calculated the power of visual attraction (PVA) according to the following formula:

$\frac{\mathrm{fixation points in}\;AOI}{\mathrm{total fixation points}}$ $÷\frac{\mathrm{pixels in}\;AOI}{\mathrm{total pixels}}$

#### 2.2.4 Heart rate assessment

We recorded participants’ heart rates before and during the observation using a portable plethysmograph monitor (Iworx IWX/404 and PT 100 model). This equipment detects slight volume changes caused by blood pulsing through the finger vessels. Pulse rate data were continuously recorded throughout the experiment and analyzed using the software provided by the company, with the average pulse rate calculated every minute. As the baseline heart rates varied in the two gardens, we also calculated the data for each individual as the change from the heart rate observed during the 3-min rest period before the viewing session.

#### 2.2.5 Mood assessment POMS2

The POMS2 Brief form (Nyenhuis et al., 1999; DiLorenzo et al., 1999; McNair et al., 1981; McNair and Lorr, 1964) was used to measure mood changes during the experiments. The form is a self-administered adjective rating scale composed of 35 items with a 0–4 score. It measures 7 mood states: anger (AH), confusion (CB), depression (DO), fatigue (FI), tension (TA), vigor (VA), and friendliness (F). The participants completed the POMS 2 Brief Form before and after observing the two gardens. As the participants’ native language was Japanese, we used the Japanese translation of the questions (Heuchert et al., 2015). Though other tests were available, we used the POMS instrument to be consistent with our own previous work in this area.

#### 2.2.6 Questionnaire

In addition to POMS2 Brief Form test, the participants were asked to answer the following questions after they had viewed the garden: (1) Do you like this garden? (2) Have you been to this garden before? (3) Do you feel relaxed after viewing this garden? (4) Do you want to come again? (5) Have you visited any Japanese gardens before? (6) If yes, how often do you visit a Japanese garden? (7) Any other comments? Subjects rated their responses to the first six questions on a 1 to 5 scale where 5 was the most positive. We had the subjects respond to the same questions both before and after their garden viewing session.

### 2.3 Procedure

**Figure 2:**
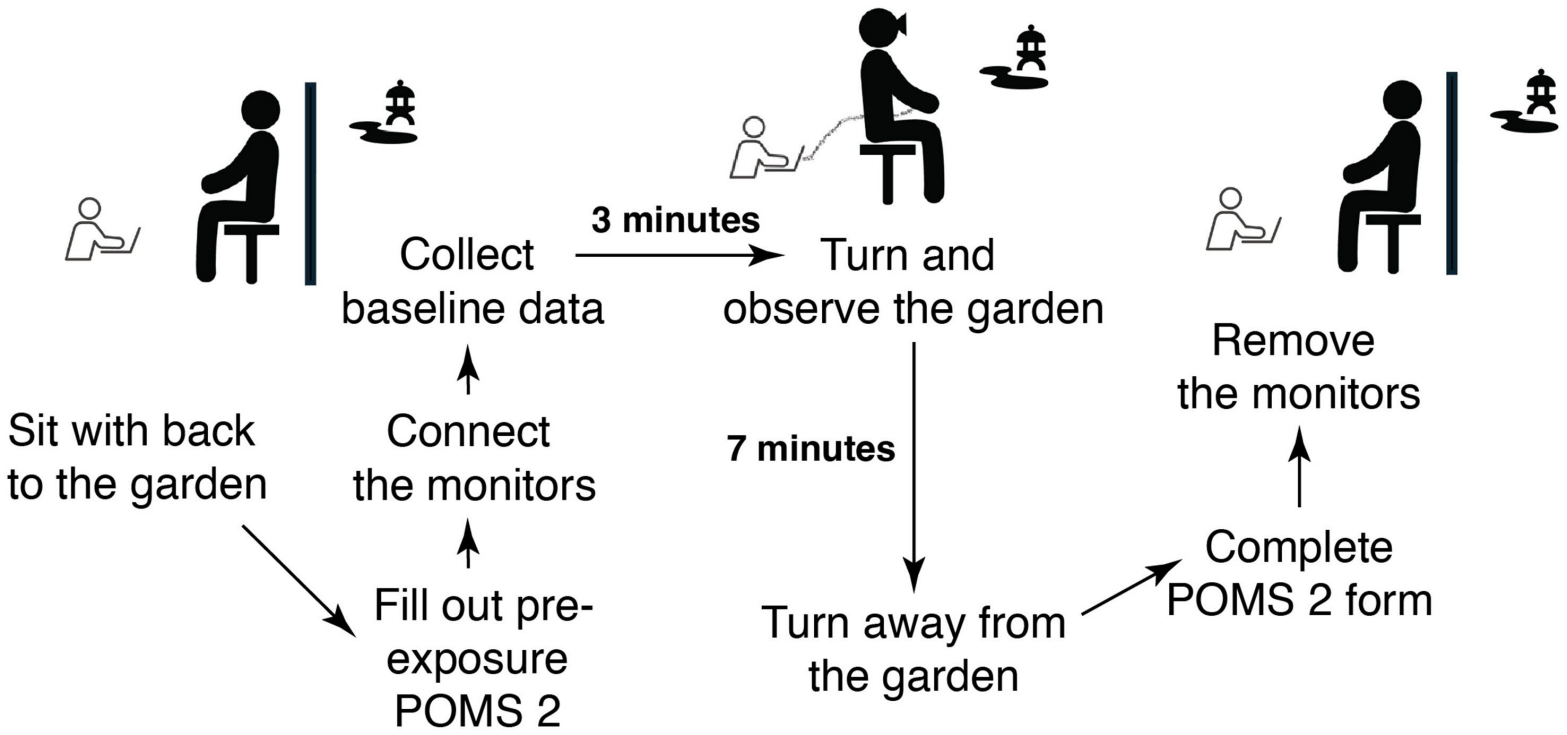
A diagram of the flow of tasks performed during each viewing session.

The experiment at Murin-an was scheduled for January 18, 2023, while the one at Kyoto University Garden (KUG) was scheduled for January 17 and 19, before and after the Murin-an experiment. Half of the participants were scheduled to observe KUG on January 18 and the other half on January 19. As discussed above the logistics of access to the Murin-an garden meant that we were restricted to a single day in January. The temperature on all 3 days was 6°C at 9:00 a.m., 12°C at 12:00 noon, and 13°C at 3:00 p.m., with partially cloudy weather and low wind speeds. Participants were provided with a warmer and blanket during their viewing of the KUG. Participants arrived at a waiting station where they filled out a consent form. They were then escorted to the garden station, which was equipped with a viewing seat, table, and movable partition to block the view while attaching and calibrating monitors. After arriving at the garden station, participants were instructed to perform the following tasks (Figure 2):

1. Participants fill out the POMS2 form while sitting with their backs to the garden.
2. The electrocardiograph and eye tracking monitor were connected to the participant by the research assistants.
3. Research assistants turn on the eye tracking monitor and adjust the camera and lighting conditions to minimize glare and ensure clear visibility of the participant’s eyes.
4. Research assistants launch calibration mode and adjust the participant’s head position.
5. Research assistants instruct the participant to look at a series of calibration points on the screen.
6. Research assistants adjust and validate the eye tracking monitors.
7. Research assistants adjust the electrocardiograph.
8. Participants rest for 3 min with no view of the garden.
9. Participants turn around and observe the garden for 7 min.
10. Participants turn around and face away from the garden.
11. Participants complete the post-exposure POMS2 form and the questionnaire.
12. Participants wait while the electrocardiograph and eye tracking monitors are removed by the research assistants.

### 2.4 Statistical procedures

We analyzed eye-movement data (Section 3.1) using SPSS (version 27.0, International Business Machines Corporation). To determine statistical significance, we used a multivariate repeated-measures analysis of variance (MANOVA) followed by Bonferroni *post hoc* tests using the mean (Prism, Graph Pad software, Version 5). The between-subjects factor was university (Kyoto U vs. Kyoto U of the Arts), and the within-subjects’ factors were garden (Murin-an, Kyoto U Garden) and timing (0–1 min, 1–2 min, 2–3 min, 3–4 min, 4–5 min, 5–6 min, 6–7 min, 7–8 min). Significance was established at *p* < 0.05 (*) and *p* < 0.01 (**). Data are presented as means ± SEM.

Heart rate data (Section 3.2) was analyzed statistically using a repeated measures ANOVA—within subject factor 1, “place” (Murin-an vs. Kyoto U Garden) and within subject factor 2, “timing” (base vs. 1 vs. 2 vs. 3 vs. 4 vs. 5 vs. 6 vs. 7).

For the analysis of the mood changes measured by the POMS2 and questionnaire (Section 3.3), we calculated the total mood disturbance (TMD) after changing the sign of the vigor (VA) and friendliness (F) score before running our calculations (since, unlike the other scores, negative scores of these are worse). We then used the change in values from “Pre” to “Post” as an indicator. A two-factor analysis of variance (ANOVA) was conducted with the remaining factors for “Student Group (art students vs. policy)” and “Location (Kyoto University Garden vs. Murin-an Garden).”

As described in more detail below, we were constrained to work with a relatively small sample size. As a result, we have in each case considered the effect size by calculating Cohen’s d-value for the various tests. The interpretation of d value can be summarized as follow

0.2: Small effect-The difference is noticeable, but small.
0.5: Medium effect-The difference is moderate and likely to be meaningful.
0.8 or above: Large effect-The difference is strong and highly meaningful.

In most cases, *d*-values are reported along with other statistical tests.

While having access to the Murin-an garden for our study was a great advantage, we are mindful of the fact that the small sample size (*n* = 16) constrains the statistical power we are able to bring to bear on our results.

## 3 Results

### 3.1 Eye movements in the two gardens

**Figure 3:**
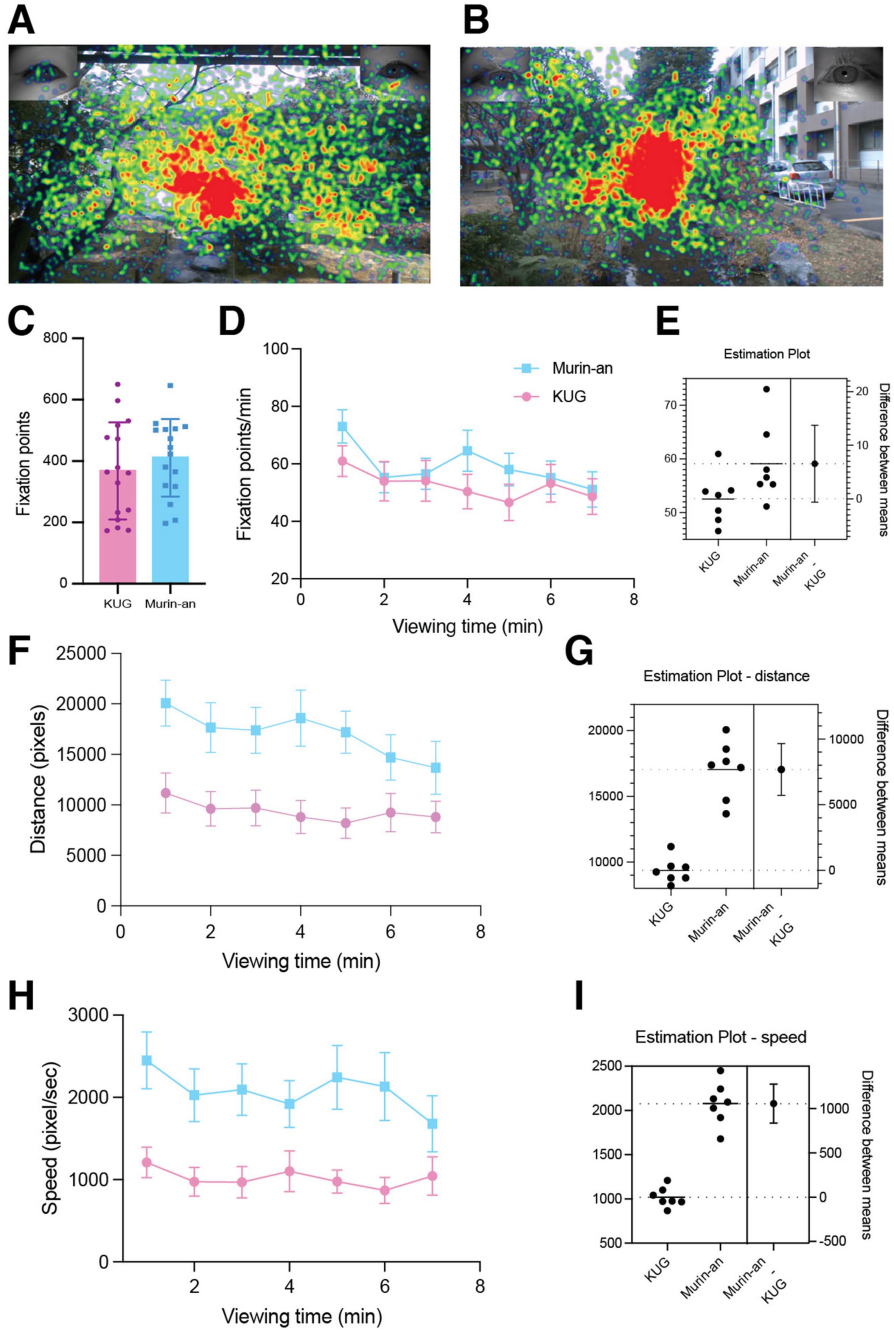
Eye movement behavior was different in the two gardens. **(A,B)** Represent heat maps of the gaze time of all 16 subject in the Murin-an **(A)** and KUG **(B)** gardens. The color scale is graded from blue (fewer fixation points) to red (more fixation points). **(C)** The average number of fixation points for all subjects in the KUG (pink) and Murin-an (blue) gardens. Error bars represent the standard error of the mean. **(D)** The average number of fixation points for all 16 subjects during each one-minute time bin (KUG—pink symbols; Murin-an—blue symbols). **(E)** Estimation statistics showing the effect size and the distribution of the differences. **(F)** The average distance traveled by the gaze during the 7-min viewing session was greater in Murin-An than in the KUG (*t*(15) = −4.30, *p* = 0.01, Cohen’s d value = 1.05). **(G)** Estimation statistics showing the effect size and the distribution of the differences. **(H)** The average speed of the participant’s eye movements was greater in Murin-an than in the KUG (*t*(15) = −4.03, *p* = 0.01, Cohen’s d value = 0.98). **(I)** Estimation statistics showing the effect size and the distribution of the differences.

We first tracked the eye movements of the participant for clues as to whether there were features of the visual field that dominated the subjects’ attention. Figures 3A,B show heat maps of the eye fixation points of all 16 participants on the 1,200 × 675 pixel picture pane. These summary maps make several clear points. The first is that the participants primarily focused on the center of the visual environment in both gardens. Second, the fixation points were far more distributed in Murin-an (Figure 3A) than in KUG (Figure 3B). The KUG garden had many of the same visual elements found in Murin-an—water, stones, trees, a bridge—but the geometric features of the building to their right may have biased the viewers’ gaze toward the center as very few of the fixation points localized to the building (Figure 3B). This potential constraint was counterbalanced by the much wider horizontal angle of view in the KUG—45.29° vs. 23.68° in Murin-an (see Methods). Despite this, when viewing the Murin-an garden the subjects’ fixation points were distributed more widely, covering the entire field of view including the edge (Figure 3A).

We noted that in the Murin-an garden, the average number of fixation points across all subjects (Table 1) was higher than in the KUG, but this difference was not statistically significant by unpaired *t*-test (*t*(30) = 0.85, *p =* 0.40, Figure 3C). Viewing the data broken down by one-minute bins gave the same result. Although the average number of fixation points was slightly higher for Murin-an compared to KUG at each point the difference was not statistically significant (Figure 3D—by unpaired *t*-test; *t*(15) = −1.82, *p =* 0.09, Cohen’s d-value = 0.44; Figure 3E by estimation statistics). Far more dramatic were the differences between the two gardens in the distance covered by the shifting gaze of the participants (Figures 3F,G), and speed of movement between fixation points (Figures 3H,I). This difference was seen from the very beginning of the 7 min viewing period, and the difference between the two curves was highly significant by both estimation statistics (Figures 3G,I) and by *t*-test for gaze distance (Figure 3F: *t*(15) = −4.30, *p =* 0.01, Cohen’s d value = 1.05) and for speed of eye movement (Figure 3H: *t*(15) = −4.03, *p =* 0.01, Cohen’s d value = 0.98).

**Table 1:** Individual eye movement data for the two gardens.

Subject number	Number of fixation points	Duration (sec)	Average fixation time (sec)
Kyoto University			
1	182	384	2.11
2	477	312	0.65
3	173	322	1.86
4	329	369	1.12
5	232	379	1.63
6	472	297	0.63
7	361	322	0.89
8	174	370	2.13
9	650	322	0.50
10	516	285	0.55
11	531	296	0.56
12	379	357	0.96
13	364	334	0.92
14	240	267	1.11
15	597	275	0.46
16	208	385	1.85
Average ± SD	368 ± 158	329 ± 40.1	1.12 ± 0.60
95% confidence limits	283–452	309–351	0.8–1.4
Murin-an			
1	207	323	0.39
2	646	280	0.43
3	197	292	1.48
4	365	306	0.77
5	317	346	1.09
6	473	380	0.80
7	501	300	0.60
8	380	377	0.99
9	503	297	0.59
10	423	336	0.80
11	512	285	0.56
12	522	269	0.52
13	444	268	0.60
14	320	323	1.01
15	505	238	0.47
16	258	268	1.04
Average ± SD	423 ± 126	308 ± 39.9	0.74 ± 0.30
95% confidence limits	346–480	284–327	0.60–0.92

**Figure 4:**
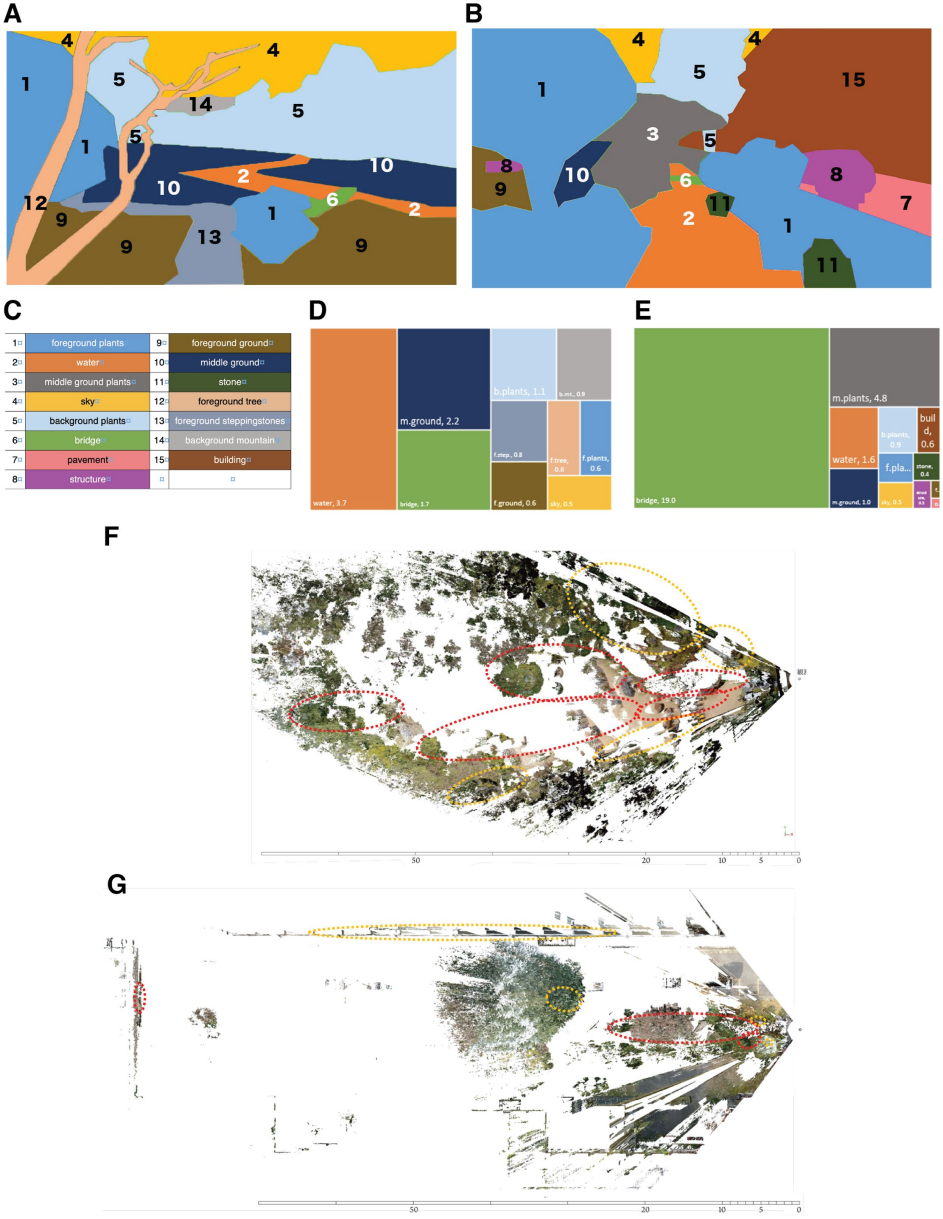
The pattern of eye gaze fixation points was different in the two gardens. We divided the visual scene in the Murin-an garden **(A)** and the KUG **(B)** based on the nature of the visual element. **(C)** The color code for the content of the areas labeled in panels **(A,B)**. **(D)** A graphic representation of the fraction of the viewing time spent gazing at each of the visual elements in the Murin-an garden. The size of each block is proportional to the fraction of the total viewing time the subjects spend gazing at that element. The numbers following the label in each block represent the average time (in minutes) that the focal point of all 16 viewers was located within that area. **(E)** A similar plot of the focal points of the 16 subjects in the KUG. **(F)** An X-Z representation of the average time spent at each focal point relative to the viewer in the Murin-an garden. The plot is the composite of the data from all 16 subjects. The dotted red lines indicate areas of high viewer interest. The dotted yellow lines indicate areas of moderate interest. **(G)** A similar plot of the data obtained in the KUG.

To further analyze the relationship between the fixation points and observed objects in the two gardens, we divided the picture pane into 15 blocks based on their content (Figures 4A,B; legend in Figure 4C). We calculated the number of fixation points in each block and normalized this value to the fraction of the entire visual field represented by that block. We termed this value the density of visual information (DVI—see Methods). On average, in the KUG (Figures 4B,E), the object most focused on by the 16 subjects was the bridge, followed by middle ground plants, and water. In Murin-an (Figures 4A,D), the most noticed blocks were the water, followed by what we termed the middle ground and the bridge. Note that these high-interest objects are arranged differently in the two gardens. In the KUG the three most-focused objects were all in the center of the visual field while in the Murin-an garden, the objects were in a more horizontal arrangement. Note also that the size or type of object did not determine its focus; instead, it was the object’s position within the overall spatial composition that guided the gaze. Most likely, in KUG, the bridge’s location at the vanishing point of the picture pane directed the gaze toward it while in the Murin-an garden, we believe that the river’s acute angle in the center directed the gaze from side to side, eventually landing on the bridge. Similar results were obtained when we analyzed the data by calculating the power of visual attraction (PVA)—the total times spent in each block (data not shown).

Our equipment and its bundled software enabled us to determine not only the 2-dimensional (X-Y) coordinates of a viewer’s gaze, but also its depth (the X-Z coordinates—Figures 4F,G). Comparing these two plots further emphasizes the dramatic differences in the ways in which the participants viewed the two gardens. In the KUG, not only did the viewer tend to focus on the center of the field in the X-Y plane, they were also focused primarily on the nearer objects in the X-Z plane (Figure 4G). The eye-movement fixation points in Murin-an were distributed throughout the background, middle ground, and foreground (Figure 4F). In the KUG, most fixation points were on the plants in the foreground, with only 2% on the background plants. In 3-dimensional space therefore, the participants in the KUG attended mostly to the lower areas and to the front and middle depths, while in Murin-an the subject’s attention tended to focus on the upper areas and far more broadly distributed through the entire depth of the visual field. These differences may have been due in part to the large Himalayan cedar tree in the KUG that blocked the view of the distant scenery, while in the Murin-an garden, as intended by its designer, there is an open view of Mt. Higashi, located several kilometers away.

### 3.2 Heart rate changes in the two gardens

**Figure 5:**
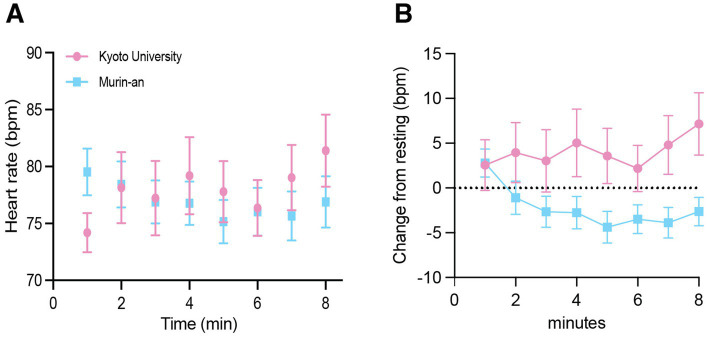
The pulse rate responds to viewing the gardens. **(A)** The average heart rate for all 16 participants during the 7-min viewing period in the KUG (pink symbols) and Murin-an (blue symbols). **(B)** The same data was normalized to the heart rate measured during the 3 min before the subject turned and viewed the garden.

Before the garden observation, participants had a 3-min resting time during which their average pulse rates were 74.2 bpm in KUG and 79.5 bpm in Murin-an (Table 2). Although this difference is notable as the participants were the same for both gardens, the reason for it is unknown. During the 7-min viewing period the average heart rate in KUG increased by 5%, while that in Murin-an decreased steadily (Figure 5A). When analyzed by repeated measures ANOVA, these differences were statistically significant (place: *F*(1, 15) = 14.43, *p =* 0.01, *η* = 0.49, 1-β = 0.94; period: *F*(7, 105) = 3.48, *p =* 0.01, *η* = 0.19, 1-β = 0.96) Meanwhile, the *interaction effect* was significantly different (*F*(7, 105) = 5.93, *p =* 0.01, *η* = 0.28, 1-β = 0.94). Figure 5B illustrates the difference in pulse rate during each minute of the viewing session relative to the rate of the same individual’s pulse rate during the resting period when they were facing away from the garden (two-tailed *t*-test, *t*(14) = 6.405, *p* = 0.0001).

**Table 2:** Individual heart rate data for the two gardens.

Time (sec)	S1	S2	S3	S4	S5	S6	S7	S8	S9	S10	S11	S12	S13	S14	S15	S16	Average
Murin-an Garden																	
0	78.91	74.40	71.96	77.93	76.10	92.08	80.87	91.09	64.62	88.66	95.82	83.48	78.54	83.53	101.6	77.41	82.31
60	80.26	80.11	70.32	77.58	74.99	83.89	74.37	81.28	61.05	71.62	88.31	82.43	78.54	81.50	96.51	72.11	78.43
120	72.35	68.71	69.94	77.70	73.67	86.14	75.17	82.68	63.06	70.65	92.56	81.50	77.49	80.38	85.03	73.12	76.88
180	77.91	70.16	73.34	75.05	73.65	81.70	76.36	80.49	60.02	68.36	88.47	83.74	77.06	81.19	89.83	71.01	76.77
240	71.29	69.39	71.72	77.45	73.79	71.43	77.56	82.27	57.54	67.89	86.69	83.08	70.80	80.17	86.44	74.90	75.15
300	68.15	65.64	70.50	75.64	73.25	86.59	78.35	81.72	59.59	72.11	88.23	83.11	73.93	78.25	90.00	71.47	76.03
360	70.76	62.22	71.82	77.50	72.06	84.81	78.45	81.36	58.81	67.46	84.59	82.55	74.28	80.61	90.66	72.28	75.64
420	73.23	66.71	69.69	73.44	72.15	82.17	78.87	85.20	61.56	67.51	88.67	79.67	73.94	80.57	97.62	79.33	76.90
Average	74.11	69.67	71.16	76.53	73.71	83.60	77.50	83.26	60.78	71.78	89.17	82.44	75.57	80.78	92.21	73.95	
Kyoto University Garden																	
0	80.82	81.23	69.36	71.04	70.35	67.99	82.65	73.04	108. 9	70.05	75.26	76.62	63.57	83.85	69.84	83.30	76.74
60	80.50	97.27	66.71	75.27	69.35	62.74	97.15	76.01	105.1	74.43	70.35	73.29	61.52	84.35	77.60	78.77	78.15
120	81.41	89.79	67.32	68.77	70.31	62.89	86.91	73.01	116.9	75.53	71.79	73.94	66.43	85.58	70.71	74.40	77.23
180	80.66	97.98	68.91	70.57	70.59	69.99	100.4	74.98	112.2	77.04	71.98	73.81	65.07	90.15	70.03	73.26	79.23
240	81.67	81.48	68.59	71.07	71.70	69.55	97.83	74.71	105.74	76.76	70.47	74.77	65.36	83.46	73.24	77.77	77.76
300	83.00	74.09	70.35	71.93	72.46	66.67	93.71	74.72	100.2	70.43	70.19	73.21	63.47	86.17	74.59	75.67	76.37
360	79.90	89.29	69.77	73.15	71.27	71.15	100.7	75.99	106.0	77.77	73.01	74.23	62.71	85.41	73.26	80.49	79.00
420	84.93	97.61	71.61	82.78	68.81	75.22	104.7	82.88	101.5	90.38	72.41	68.43	63.93	90.06	73.17	73.14	81.35
Average	81.74	88.59	69.08	73.07	70.61	68.27	95.50	75.67	107.1	76.55	71.93	73.54	64.01	86.13	72.81	77.10	

We next asked whether we could detect changes in heart rate in response to a subject’s viewing a particular element in the visual field, for example the bridge or the stream. This analysis is made difficult because the time scales of eye movements (milliseconds) and heart rate (minutes) differ by several log units. We reformatted the heart rate data to provide an estimate of beats per minute (bpm) by sampling the number of beats every 6 s. On average this would represent only six beats so that one extra or one fewer pulses would substantially affect the calculation. We next defined regions of the visual field that corresponded to specific garden elements and identified the times when a participant’s gaze lingered in that region as an area of interest or a focal point. We tallied the frequency of a participant’s focus on each object in each six-second time bin then overlaid these results with the six-second heart rate estimates. We found no apparent correlation between any one object in the visual field and a change in the heart rate (Supplementary Figure 2).

### 3.3 Mood changes—POMS2 and questionnaire responses

**Figure 6:**
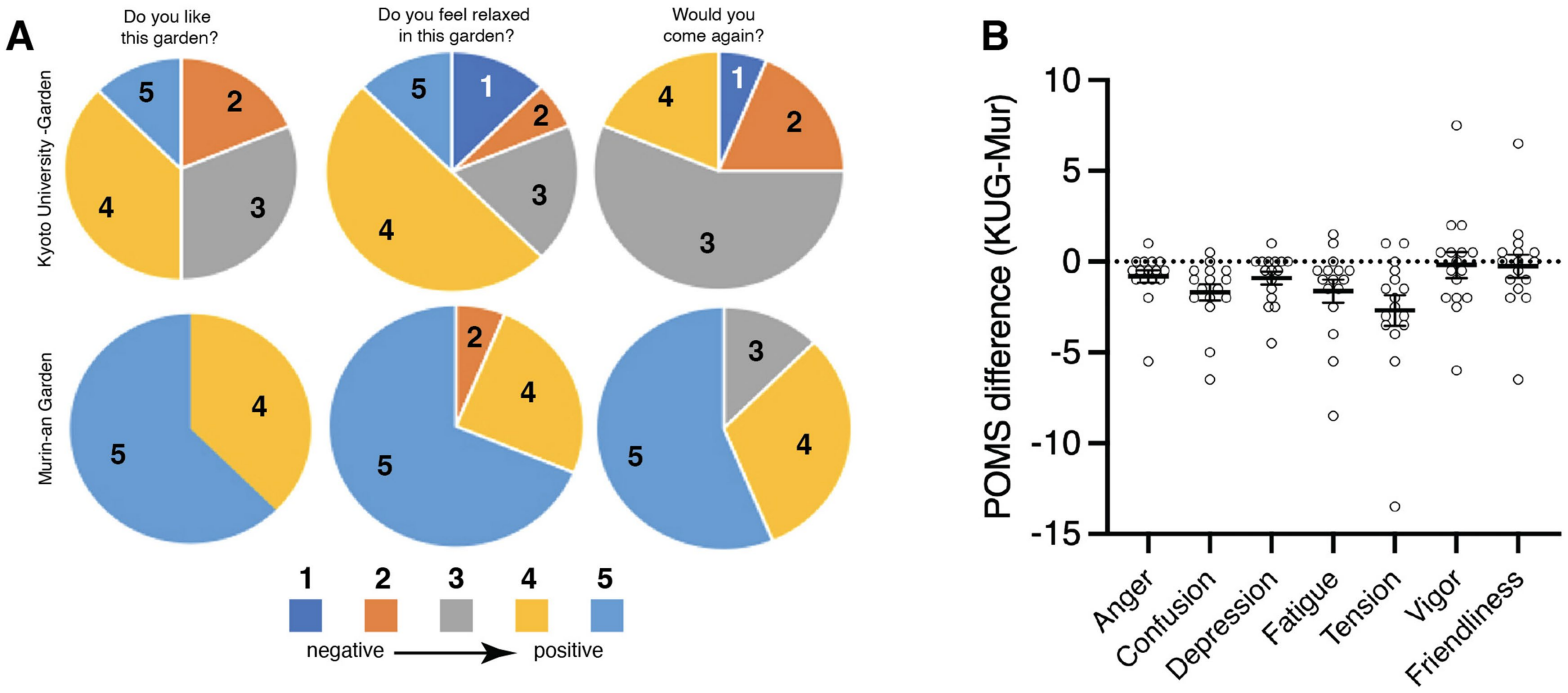
The response of the mood of the viewers to questionnaire and the POMS. **(A)** Pie charts showing the percentage of respondents who assigned that score to their answer. The legend at the bottom illustrates the meaning of score. The number in each pie wedge is the score assigned to that wedge. **(B)** The results of the POMS instrument before and after garden viewing. The X-axis represents the seven categories of mood, the Y-axis represents the difference of the differences (see text for details). The mostly negative scores of this metric indicate that the improvement of mood in Murin-an was greater than that observed in the KUG.

The two gardens elicited very different qualitative responses as well. Participants’ responses to a simple questionnaire (see Methods) showed that they liked (*t*(15) = 4.86, *p* = 0.01, Cohen’s d value = 1.18), felt relaxed in (*t*(15) = 5.92, *p* = 0.01, Cohen’s d value = 1.44), and wanted to revisit (*t*(15) = 6.33, *p* = 0.01, Cohen’s d value = 1.58) the Murin-an garden significantly more than the KUG (Figure 6A). The POMS test results in the two gardens also differed. To determine whether viewing the garden impacted mood, we administered the POMS2 both before and after participants viewed the two gardens. The scores of the first test (administered before the garden was viewed) varied considerably among the participants. We calculated the responses of each individual participant as the difference between their responses on the second test (after viewing) minus their responses on the first (before viewing). We then compared the magnitude of this after-minus-before difference in the KUG with the magnitude of the difference in the Murin-an garden (Figure 6B). In the POMS2, a positive value in anger (AH), confusion (CB), depression (DO), fatigue (FI), and tension (TA), and a negative value in vigor (VA), and friendliness (F) indicates an improvement in mood. Therefore, if Murin-an is more effective in improving anger (AH), confusion (CB), and depression (DO) than KUG, then the value of KUG—Murin-an will be negative. Moreover, if Murin-an is more effective in improving vigor (VA) and friendliness (F) than KUG, then KUG—Murin-an will be positive. As represented in Figure 6B, for the first five measures of mood, the difference in Murin-an was negative, and for measures of the vigor and friendliness components of mood, there was virtually no change in either garden (not shown). Furthermore, using the Total Mood Disturbance (TMD—see Methods) a two-way ANOVA of the results reveals a significant difference between the two gardens (*F*(1, 14) = 11.28, *p* = 0.01). Overall, we can say that the Murin-an garden had a stronger effect on the POMS2 score than the KUG.

### 3.4 Impact of student background

The backgrounds of the 16 participants differed in ways that led us to ask whether these differences affected their responses to the garden in any consistent way. The seven students from Kyoto University were from a program in policy and city planning. While they were familiar with the importance of green spaces, of the seven, two had never visited a Japanese garden, four visited a few times a year, and only one visited a few times a month. By contrast, all nine participants from Kyoto Art University were studying to become Japanese garden specialists and had taken classes on Japanese gardens at the university. Seven reported that they visited Japanese gardens several times a month; two reported visiting a few times a week. Further, as it was on their campus, all seven policy/city planning students had seen the KUG previously, but only two had been to Murin-an before the experiment. By contrast, of the 9 art students, only one had visited the KUG, but four had previously visited Murin-an.

**Figure 7:**
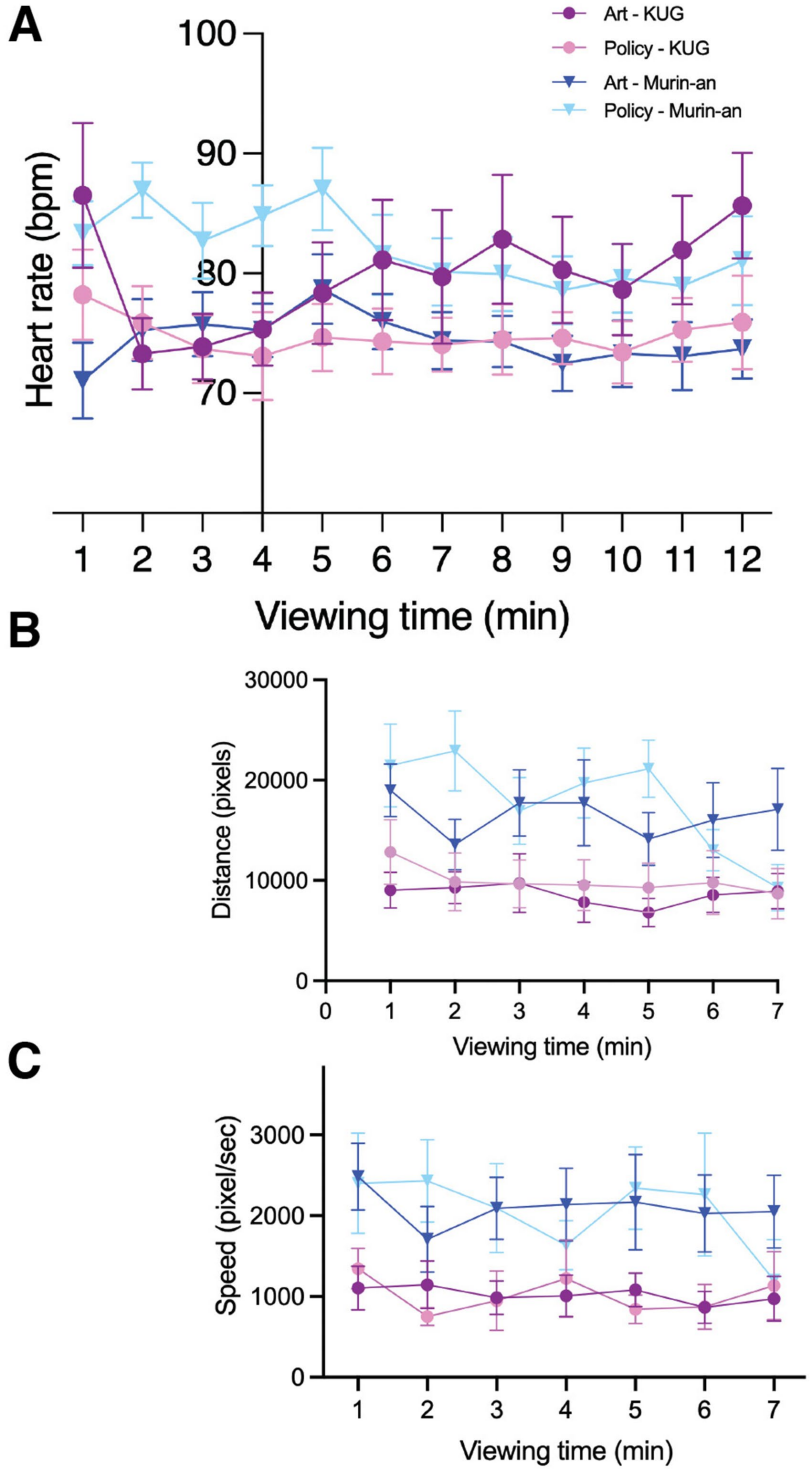
The background participants affected their responses to the two gardens. **(A)** The heart rate data are plotted separately for the policy/planning and art students. The responses differ between the two groups (details in the text). **(B)** The average distance traveled by the gaze of the 16 participants was similar between the two groups, but both groups showed different responses to the two gardens. **(C)** The average speed of the shift in gaze was similar between the two groups, but both groups showed different responses to the two gardens.

Each of the four groups showed a unique heart rate response to the gardens. Once they had settled down during the pre-viewing period in the KUG, the heart rates of the students with a background in Japanese garden techniques climbed steadily throughout the garden viewing session (Figure 7A, dark purple). This was suggestive of an increased level of stress/irritation. In the Murin-an garden, these same students had an initial rise in their heart rate during the first minute of viewing followed by a slow but steady decline in rate (Figure 7A, dark blue). The policy/city planning students, with less previous exposure to Japanese gardens, responded quite differently. In the University setting, they were quicker to settle down during the pre-viewing period and once they were allowed to view the KUG, their heart rates remained virtually unchanged during the viewing period (Figure 7A, pink). In the Murin-an garden, their resting rate was noticeably higher than either their own rate before viewing the KUG or the rate of the art students in Murin-an. Despite this suggestion of elevated stress/anxiety, once the viewing period began, their heart rates also decreased and remained low throughout (Figure 7A, light blue). Overall, we noticed that the absolute heart rate could vary but the relative change over the viewing period was consistent in the two different groups—declining more in the Murin-an garden than in the KUG. We also asked whether the viewing strategies of the two groups—total eye movement distance and speed of movement—differed in the two different gardens. From minute to minute there were more noticeable fluctuations in the distance traveled (Figure 7B) and speed of the eye movements (Figure 7C) in the Murin-an garden in both groups of students, compared with the KUG where both measures of gaze remained relatively stable and constant during the viewing period.

The qualitative observations also differed between the two groups, but not dramatically. In Murin-an scores for the question about feeling relaxed by viewing the garden increased by 124% among the city planning students and 140%, among the art students compared to KUG. Similarly, the scores for the question about if they want to revisit the garden increased by 122 and 153% among planning and art groups, respectively in Murin-an compared to KUG (Figure 7). Overall, Murin-an was more popular than KUG among all participants, with art students showing a stronger preference for Murin-an than the city planning students. We performed a 2-way ANOVA (two gardens, pre-post) followed by Bonferroni *post hoc* tests for the POMS2 results of the two groups. For the art students, we found a significant difference between the pre and post values for both gardens (*F*(1, 8) = 6.98, *p* = 0.017, *η* = 0.532, 1-β = 0.753) but no significant difference between the pre-viewing values of the two gardens (*F*(1, 8) = 2.482, *p* = 0.154, *η* = 0.237, 1-β = 0.285). For policy/planning student, we also found a significant difference between the pre and post values for both gardens (*F*(1, 8) = 9.291, *p* = 0.016, *η* = 0.537, 1-β = 0.761), with a significant difference between pre and post values in Murin-an (pre: 14.899 ± 2.951 vs. post: 3.444 ± 3.614, *p* = 0.001, *η* = 0.622, 1-β = 1.000) in post hoc tests, but no change in the KUG (pre: 13.444 ± 6.039 vs. post: 4.556 ± 3.056, *p* = 0.123, *η* = 0.164, 1-β = 0.324). In short, the art students’ mood improved in both gardens, while policy students’ mood improved significantly only in Murin-an.

## 4 Discussion

Our findings provide several important advances in our ongoing studies of Japanese gardens and their effect on the viewer. Our early work in this area focused on elderly participants, including those with advanced dementia (Goto and Fritsch, 2011; Goto et al., 2014; Goto et al., 2013). These studies revealed that there were features in the elements and design of a Japanese style garden that were unique in their ability to engage participants and lower both their qualitative sense of tension and its physiological manifestation in heart rate. Perhaps most intriguing was how viewing the garden cut through the fog of dementia and produced effects, even in participants whose MMSE (Mini-mental status exam) scores were below 15 (Goto et al., 2013). The robust nature of the effect led us to explore its neurobiological underpinnings, which in turn led us to an interest in tracking eye movements (Liu et al., 2020; Goto et al., 2020; Goto et al., 2017). Our hypothesis was that, by correlating the object of a participant’s attention with the momentary change in their heart rate, we could identify those features of the garden—a stone lantern, a water element, a specific type of plant—that were most responsible for eliciting the calming effect we had documented. The results, however, did not support this idea. Rather, the data suggest that it was the overall pattern of eye movements that differed most significantly when participants viewed a Japanese style garden (Goto et al., 2020).

The current study offers a possible explanation as to why our initial hypothesis was incorrect, and ironically, it may have been the uncomfortable nature of the viewing environment that was the key to this insight. We chose to measure the effect of the Murin-an garden because it is a classic and highly regarded design that we hoped would maximize the signal we were seeking (a more detailed description of its history and design principles can be found in the Supplementary Figure 1). Unfortunately, it is also a popular tourist attraction and because of this, our access for the purpose of experimentation was restricted to a single day in January. Kyoto winters are cold and the villa from which the garden was viewed was not heated. The KUG viewing spot was completely outdoors and the viewing sessions were held during the same January days. We provided blankets for warmth at both locations, but most participants remarked about the conditions. The wintertime viewing schedule also meant that both gardens were devoid of any flowering plants and their deciduous trees were bare. The subdued colors meant that the participants were influenced primarily by the design features of the two gardens.

The pulse rate measurements show the effects of this minimalist stimulation. In previous work, in comfortable conditions, even a less than professional Japanese style garden was sufficient to induce a perceived and physiologically detectable calming effect (Goto et al., 2013). In this study, despite the cold winter weather, all 16 participants experienced significant physiological relaxation while observing the Murin-an garden. Although only some Kyoto Art University students reported appreciating the scenery of Murin-an in the questionnaire, the results from the heart rate and POMS2 evaluation revealed that the Kyoto University students, who had less knowledge of Japanese gardens than the Kyoto Art students, nonetheless experienced greater physiological relaxation and mood improvement in Murin-an. This suggests that the relaxation effect of Murin-an is not only much greater than that of a typical Japanese garden but also more effective for first-time viewers. This is consistent with the idea that a certain pattern of eye movements can induce a relaxing effect regardless of the subject’s background.

Yet viewing the KUG led to no significant drop in heart rate, although the POMS2 revealed that most measures of mood improved. Despite the cold, however, the Murin-an garden was highly effective and produced a calming effect on both heart rate and mood. Both groups of students experienced an increase in their heart rate during the first minute in Murin-an after which the policy/city planning students showed a steeper decline in heart rate while the art students heart rate decreased more slowly. In other words, viewing Murin-an had a greater calming effect on the participants who had less knowledge of Japanese gardens. This suggests that the relaxation effect of Murin-an is much higher than that of a typical Japanese garden and also more effective for first-time viewers.

One caveat that should be considered in evaluating the data is that we did not collect a detailed medical history of the students or ask whether they were taking any medications. As all the subjects were students, their daily activities were likely similar, but the possibility exists that our subjects had intrinsic differences in baseline stress that might have influenced their individual responses. That said, the POMS2 is a detailed questionnaire designed to measure mood and stress that we administered before and after the viewing sessions. We found no evidence that any of our subjects had unusual scores on the first test. Also, the distribution of the heart rates was fairly narrow (e.g., Figures 1, 4, 7), and there were no examples of erratic heart activity during any of our monitoring sessions. While not definitive, data such as these suggest that intrinsic health differences were not an important factor in evaluating our data.

The eye-tracking data add an important dimension to the analysis. We found a substantial difference in the gaze movements during the viewing sessions of the two gardens. To some extent, a participant viewing a garden they had never seen before led them to take in a wider range of views. While this may have been a factor for some participants, this effect was minor compared to our observation that the range of fixation points in all three planes, the distance traveled by the eyes, and the speed with which they moved were all significantly greater in Murin-an than in the KUG. As we observed previously (Goto et al., 2020), the participants’ gaze in both gardens tended to concentrate in the center of the visual field. Nonetheless, the distribution of points was far broader in Murin-an, extending to the edge of the visual field especially in the horizontal direction. In the KUG, the regular geometry of the windows in the building at the right edge of the visual field did not draw the participants’ attention and may have served to “repel” gaze and thus help to keep it centered in the visual field. Compounding this effect, the vertical lines of the cedar tree and other large plants would discourage the viewer from moving their gaze in the center of the field. Whatever the actual causes, the result was that in Murin-an, but not in the KUG, the participants’ gaze shifted in the horizontal direction more often and more rapidly.

We note the analogies between our findings and the principles used in a technique known as eye-movement desensitization and reprocessing (EMDR). EMDR was developed by psychologist Francine Shapiro who is credited with first noting the association between eye movement and mood (Shapiro, 1989; Shapiro et al., 1994). The theory of the method is to reduce stress by providing bilateral stimulation through the manipulation of eye movement during the recollection of a specific memory. After its initial discovery other sensory domains such as sound and touch have been added (Shapiro, 1999). The technique is widely used, especially to treat the persistent stress associated with traumatic events (e.g., PTSD), but its neurological basis is not understood (Pierce et al., 2023; Valiente-Gomez et al., 2017; Zarghi et al., 2013). The success of the technique has been suggested to be due in part to the strong bilateral nature of the visual stimuli used and some authors have noted its relationship to the REM phase of sleep (Stickgold, 2002). Although the exact neuroanatomical pathways that underlie the phenomenon are still under study (Vereecken and Corso, 2024), work in mice has drawn attention to the superior colliculus as important in the reduction of fear by bilateral stimulation (Baek et al., 2019). Pathways to the adrenal gland from motor areas of cortex and the eye-movement regions of cerebellum may also play a role (Dum et al., 2019; Seese et al., 2023).

Caution is needed in adopting this analogy. The EMDR technique specifically pairs bilateral stimulation with recall of a traumatic memory. No such pairing is evident in our experiment. Similarly, an individual viewing a garden from a single vantage point with no outside instruction about how they should focus their view is different from a session of EMDR where the stimulation is highly controlled by the person administering the therapy. Despite these differences, the work in EMDR and our own observations align in ways that offer suggestions for future exploration. The strong effect of bilateral visual stimulation that is achieved during EMDR suggests that it is the wider eye movement patterns that are elicited in a well-designed garden such as Murin-an that are the reason for the physiological and psychological effects that we observe. Thus, our search for a specific item in the garden that acts as the source of relaxation was always going to fail. Instead, it suggests it is the totality of the design, and the rapid and broad eye movements evoked, that are the key ingredients. The later EMDR work of Shapiro and others showing the effectiveness of other sensory modalities urges more consideration of the sonic and even olfactory environment of a garden in eliciting the full stress reduction effects that we observe.

## 5 Conclusion

Our study adds depth to our understanding of how garden viewing, a simple, non-pharmacological intervention, can reduce both physiological and psychological evidence of stress. The strengths of the work include, first and foremost, our ability to negotiate access to a world-class Japanese garden to test the effects of its design principles on a viewer. A second important strength is the multi-dimensional nature of the analysis—the combination of eye tracking, pulse rate measurements, and the POMS instrument to monitor subject responses. The results both confirm and extend our previous work in the area and the analogies with EMDR offer important new avenues for future exploration. There are weaknesses in the work, however, that should also be acknowledged. The small sample size, while unavoidable, limits the statistical power of our results. The mostly homogeneous nature of our subjects (young students of similar ethnic backgrounds) means that additional work is required to assure the generalizability of our findings. In designing these studies it will also be of interest to test the persistence of the stress reduction after a person leaves the garden. Despite these caveats, our findings make it clear that the relaxation effect of the Murin-an garden is not only much greater than that of a typical Japanese garden but also more effective for first-time viewers. This is consistent with the idea that a certain pattern of eye movements can induce a relaxing effect regardless of the subject’s background.

## Data Availability

The original contributions presented in the study are included in the article/Supplementary material, further inquiries can be directed to the corresponding authors.
