# Supplementary material for: Eye movement patterns drive stress reduction during Japanese garden viewing
Source: Front Neurosci. 2025 May 15;19:1581080. doi: 10.3389/fnins.2025.1581080 (PMC12119466; doi:10.3389/fnins.2025.1581080)
Supplement: Supplementary file 2 [file Data_Sheet_2.pdf]

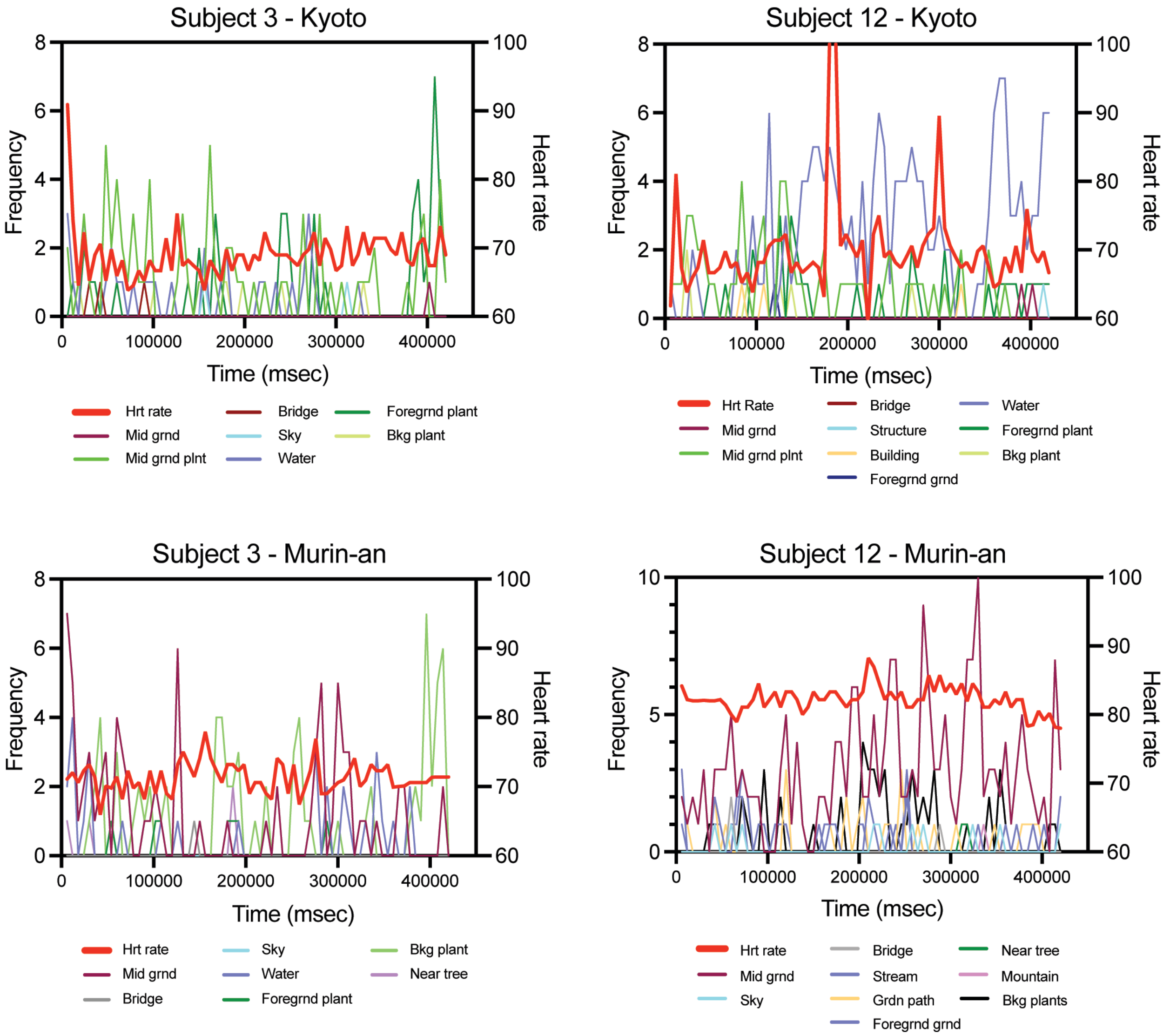

Supplemental Figure 2. **Lack of correlation between the object in view and changes in heart rate**. The number of times an object was viewed during a 6-second time bin (frequency, left Y-axis) and heart rate were estimated based during the same 6-second bin. Two representative subjects are shown in each of the two gardens.
